# Nexus of Stochastic and Deterministic Processes on Microbial Community Assembly in Biological Systems

**DOI:** 10.3389/fmicb.2019.01536

**Published:** 2019-07-04

**Authors:** Heyang Yuan, Ran Mei, Junhui Liao, Wen-Tso Liu

**Affiliations:** Department of Civil and Environmental Engineering, University of Illinois at Urbana–Champaign, Urbana, IL, United States

**Keywords:** microbial ecology, community assembly, neutral theory, stochastic processes, deterministic processes

## Abstract

Microbial community assembly in engineered biological systems is often simultaneously influenced by stochastic and deterministic processes, and the nexus of these two mechanisms remains to be further investigated. Here, three lab-scale activated sludge reactors were seeded with identical inoculum and operated in parallel under eight different sludge retention time (SRT) by sequentially reducing the SRT from 15 days to 1 day. Using 16S rRNA gene amplicon sequencing data, the microbial populations at the start-up (15-day SRT) and SRT-driven (≤10-day SRT) phases were observed to be noticeably different. Clustering results demonstrated ecological succession at the start-up phase with no consistent successional steps among the three reactors, suggesting that stochastic processes played an important role in the community assembly during primary succession. At the SRT-driven phase, the three reactors shared 31 core operational taxonomic units (OTUs). Putative primary acetate utilizers and secondary metabolizers were proposed based on K-means clustering, network and synchrony analysis. The shared core populations accounted for 65% of the total abundance, indicating that the microbial communities at the SRT-driven phase were shaped predominantly by deterministic processes. Sloan’s Neutral model and a null model analysis were performed to disentangle and quantify the relative influence of stochastic and deterministic processes on community assembly. The increased estimated migration rate in the neutral community model and the higher percentage of stochasticity in the null model implied that stochastic community assembly was intensified by strong deterministic factors. This was confirmed by the significantly different α- and β-diversity indices at SRTs shorter than 2 days and the observation that over half of the core OTUs were unshared or unsynchronized. Overall, this study provided quantitative insights into the nexus of stochastic and deterministic processes on microbial community assembly in a biological process.

## Introduction

Microbial community is shaped by stochastic and deterministic processes (Ofiţeru et al., 2010), but the extent to which these two processes influence community development is still a much debated topic (Ayarza and Erijman, 2011; Valentín-Vargas et al., 2012; Zhou et al., 2013). For stochastic processes, neutral theory predicts that communities are randomly assembled through birth-death, drift, and speciation (Sloan et al., 2006; Zhou and Ning, 2017). For deterministic processes, community assembly is thought to be governed by interspecies interactions (e.g., competition, predation, and syntrophy) and niche differentiation (e.g., operational conditions in bioreactors) (Wang et al., 2013; Ju et al., 2014).

The study of ecological succession can help understand the microbial community assembly mechanisms. Previous studies have focused on the effects of stochastic processes during microbial succession in natural ecosystems including soil (Ferrenberg et al., 2013), groundwater (Zhou et al., 2014), and salt marsh soil (Dini-Andreote et al., 2015). Those studies consistently demonstrate that the succession of microbial community is initially driven by stochastic processes, and progressively determined by environmental factors such sodium concentration and soil organic matter (Dini-Andreote et al., 2015). It is reasonable to hypothesize that stochastic and deterministic processes can interact with each other and together shape the community structure. The hypothesis is supported by the findings from natural ecosystems that deterministic disturbance may promote random community assembly (Didham et al., 2005). Strong disturbances such as wildfire can reset assembly processes by exerting adverse impacts equally on all members in a microbial community, thus creating a pristine environment in which stochastic processes briefly govern community assembly (Ferrenberg et al., 2013).

Engineered biological systems allow the manipulation of individual deterministic factors and can be used as a model system to elucidate community assembly mechanisms (Daims et al., 2006; Wells et al., 2009; Yang et al., 2011; Vanwonterghem et al., 2014). Among various deterministic parameters, sludge retention time (SRT, the time that microorganisms stay in a bioreactor) represents a strong driving force of the community dynamics (Kim et al., 2011). Microbes with low growth rates will be washed out if short SRTs are imposed, leaving fast-growing microbes in the dominant populations. This was evidenced by the drastic decline in diversity and significant change in community composition when the SRT of a wastewater treatment plant (WWTP) was reduced from 30 to 3 days (Vuono et al., 2014). The impacts of SRT was further inferred by an environment-species network constructed based on the communities from a full-scale WWTP (Ju and Zhang, 2015).

Modeling-based studies suggest that the combination of stochastic and deterministic processes can better explain the community structure variation in engineered systems (Ofiţeru et al., 2010; Curtis et al., 2013). This incorporative theory is supported by experimental observations. For instance, a randomly assembled microbial community shifted to niche-based communities when the deterministic selection imposed by wastewater concentration became stronger (Van Der Gast et al., 2008). The interactive effects of stochastic and deterministic processes on community assembly has not been reported in engineered biological systems, but is possibly present due to the ubiquitous impacts of stochastic processes.

The objectives of this study are to characterize stochastic processes during succession at the start-up phase, to understand the deterministic effects of SRT on community dynamics and compositions, and to quantify the interactive impacts of stochastic and deterministic processes on microbial community assembly. To achieve these objectives, triplicate lab-scale activated sludge reactors were seeded with identical sludge inoculum and operated in parallel. The reactors were operated under eight different SRTs by step-wise decreasing the SRT from 15 days to 1 day over 270 days. The microbial community was characterized using 16S rRNA amplicon sequencing and the succession steps at the start-up phase were identified using hierarchical clustering. SRT-driven dynamics were further examined with K-means clustering, network analysis and the synchrony of the core populations across reactors. Finally, the Sloan’s Neutral Community Model and a null model were applied to determine the relative contribution of stochastic vs. deterministic processes.

## Materials and Methods

### Reactor Operation

Three activated sludge reactors were built using acrylic plastic. The total volume was 1.85 L and the working volume was maintained at 1.5 L. The reactors were inoculated with 11.25 g volatile suspended solids (VSS) collected from the aeration tank of a municipal WWTP, and fed with synthetic wastewater that consist of (per liter): CH_3_COOH, 5.0 ml; CH_3_COONa⋅3H_2_O, 7.8 g; peptone, 2.6 g; and CaCl_2_⋅2H_2_O, 0.7 g; MgSO_4_⋅7H_2_O, 4.5 g; (NH_4_)_2_SO_k_ 0.6 g; and KH_2_PO_4_, 0.35 g (Liu et al., 1994). The seed was collected in February, when the WWTP was operated at an average temperature of 9.7°C and an SRT of 2.8 days.

The reactors were operated at room temperature under a fed-batch mode with four 6-h cycles per day (Supplementary Figure S1). Each cycle included: 5-h aeration, 2-min sludge withdrawing, 45-min settling, 6-min supernatant withdrawing, 2-min substrate feeding, and 5-min tap water replenishing. The SRT (15, 10, 7, 5, 3, 2, 1.5, and 1 day) was controlled by varying the volume of the withdrawn sludge. The operation period was approximately five times of each SRT (e.g., 85 days for 15-day SRT) to ensure sufficient time of community establishment, and the total operation time was 270 days. The hydraulic retention time was decoupled from SRT by withdrawing the supernatant and replenishing tap water. The hydraulic retention time and organic loading rate were maintained at 18 h and 0.6 g chemical oxygen demand (COD) L^-1^ d^-1^, respectively, which was typical for a real WWTP. Soluble COD was measured used 0.22 μm membrane filters according to the manufacturer’s instruction (DR/890, Hach, CO, United States). VSS were measured according to standard methods (APHA, 1985).

### DNA Extraction and 16S rRNA Gene Amplicon Sequencing

Each reactor yielded 96 samples (total 288 samples) over 270 days of operation. Samples were stored immediately at -80°C before DNA extraction. Sequencing of 16S rRNA gene amplicon were performed as described preciously (Mei et al., 2017). Briefly, genomic DNA was extracted using FastDNA SPIN Kit for Soil (MP Biomedicals, Carlsbad, CA, United States), and PCR amplification was performed with the universal primer sets 515F/909R. The pooled PCR products were sequenced on an Illumina Miseq Bulk 2 × 300 nt pared-end system at the Roy J. Carver Biotechnology Center at the University of Illinois at Urbana–Champaign. Sequencing data are available in GenBank under the accession number PRJNA478774.

### Community Analyses

Paired-end sequences were assembled and denoised and OTUs were picked using the QIIME 2 plugin DADA2 with the following parameters: –p-trunc-len-f 300, –p-trunc-len-r 220 (Caporaso et al., 2010; Callahan et al., 2016). The positions to truncate were determined from the summary of the QIIME 2 demultiplexing results. Taxonomy was assigned using the QIIME 2 plugin feature-classifier and Greengenes database as the reference, with the classifier trained on 97% OTUs (Mcdonald et al., 2011; Bokulich et al., 2018). Unweighted pair group method with arithmetic mean (UPGMA) based on unweighted UniFrac distance was performed in QIIME 2. Phylogenetic tree was constructed using the methods of neighbor joining and parsimony provided in ARB program (Ludwig et al., 2004). Permutational Multivariate Analysis of Variance (PERMANOVA, *N* = 999), α-diversity indices (Chao1, Shannon, and Simpson), two-sample *t*-test and Analysis of Variance (ANOVA) were performed using R. A *p*-value of 0.05 was used to identify significant difference. The total 288 samples resulted in 10,955,415 high quality sequences. After removing singletons, 2,244 OTUs (similarity = 97%) were obtained for the three reactors. The plateaus of the rarefaction curves indicated the capture of the dominant species in the communities (Supplementary Figure S2).

Major OTUs were selected when the relative abundance was ≥0.5% in at least one sample (Ling et al., 2018). To perform succession analysis, the samples at the start-up phase (SRT 15 days) were hierarchically clustered using Kendall’s τ similarity metric (Koenig et al., 2011). To understand the interactive effects of stochastic and deterministic processes on community assembly, core populations at the SRT-driven phase (SRT shorter than 10 days) were identified. For each OTU, the average relative abundance and frequency at a given SRT were calculated. Core populations were then selected when the SRT-specific abundance and frequency were ≥0.75% and ≥75%, respectively, at any SRT (Hanski, 1982). Unlike previous studies, in which one core population was identified from multiple reactors (Mei et al., 2016; Saunders et al., 2016), here core population was defined for each reactor to facilitate the discussion on community assembly mechanisms. For core OTUs that were shared by all three reactors, their abundances were averaged and normalized to the maximum, and K-means clustering was performed to categorized them into seven groups based on the seven SRTs at the SRT-driven phase (Eiler et al., 2011). The shared core OTUs were also used for network analysis with CoNet (Detti et al., 2011). To correct for multiple testing issue, Spearman’s correlation was calculation by combining permutation (*N* = 999) and bootstrap, and *p*-value was adjusted by the Bonferroni correction. Each edge represented significant correlation (*p* < 0.05) with a Spearman’s rho ≥ 0.6. Finally, the synchrony of the core OTUs in the three reactors was evaluated pair-wise using Spearman’s correlation (Griffin and Wells, 2016).

### Neutral Model Analysis

The potential importance of stochastic processes was evaluated using Sloan’s Neutral Community Model (Sloan et al., 2006), which predicted the relationship between the frequency of each OTU in a set of local communities (i.e., a given SRT in a reactor) and their abundance in a meta-community (i.e., all eight SRTs in a reactor). The SRT-specific frequency and average relative abundance of all OTUs in a certain reactor were fitted by the estimated migration rate, *m*, as previously described (Burns et al., 2015; Ling et al., 2018). The fitting was implemented using non-linear least-squares fitting with the R package minpack.lm (Elzhov et al., 2010). The goodness-of-fit (generalized *R*^2^) and estimated migration rate (*m*) of the triplicate reactors were averaged.

A null model analysis was also performed to calculate the deviation of null expectations from actual observations and thereby quantifying the contribution of stochastic processes to community assembly (Zhou et al., 2014). Briefly, the number of species (α) at a given time point in a reactor was counted. Next, α species were randomly drawn from the original time point and reactor. The drawing was repeated 1,000 times. The Jaccard’s similarity between two reactors was calculated after each iteration and average expected Jaccard’s similarities (*J*_exp_*_,i_*) at each time point *i* was obtained from the 1,000-time drawing. The contribution of stochasticity to community assembly was calculated as:

$$\%stochasticity=1-\frac{j_{obs,i}-j_{exp,i}}{j_{obs,i}},$$

where *J*_obs_*_,i_* is the actual Jaccard’s similarity between two reactors at the time point *i*. The pair-wise contribution of stochasticity of the triplicate reactors was averaged. Meanwhile, the abundance of individual OTU at each time point was averaged based on the 1,000-time drawing to construct a null community. Permutational analysis of multivariate dispersions (PERMDISP) was conducted to examine the significance of difference between the actual and the null communities in each reactor. The R script can be found in the Supplementary Material.

## Results

### Differences at the Start-Up and SRT-Driven Phases

**FIGURE 1:**
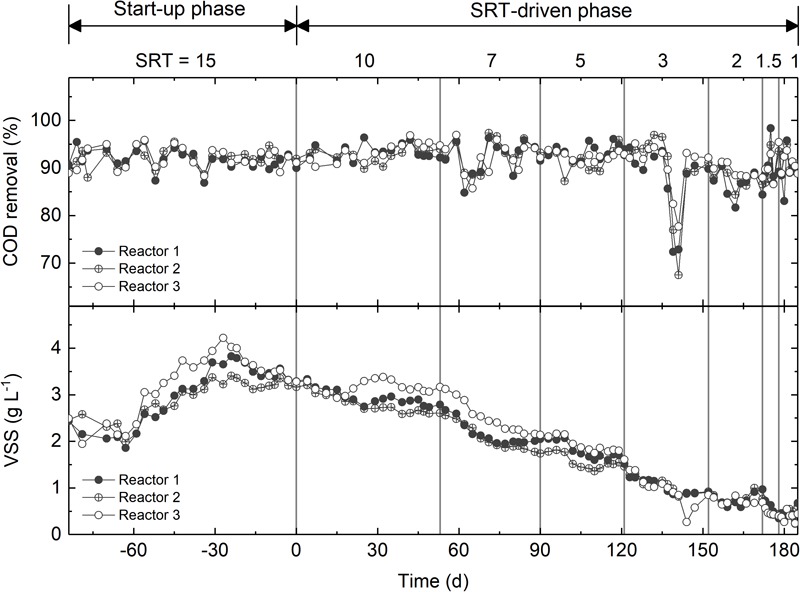
Chemical oxygen demand removal and VSS of the three reactors.

The three activated sludge reactors were fed with synthetic wastewater with acetate as the main carbon source. During the start-up phase, the reactors were operated under an SRT of 15 days for 84 days. The COD removal efficiency was consistently higher than 90% (Figure 1). The VSS, which was used as a proxy of biomass concentration, decreased slightly from 2.5 to 2.0 g L^-1^ within the first 3 weeks, then increased to approximately 3.8 g L^-1^, and eventually reached a steady state of 3.5 g L^-1^ (Figure 1). After start-up, the SRT of all reactors was step-wise reduced from 10 days to 1 day. During this SRT-driven phase, all three reactors consistently exhibited high COD removal efficiency (Figure 1). The sudden drop in COD removal between days 222 and 229 was caused by pump failure, and was recovered rapidly. The VSS was strongly affected by the changes in SRT, and decreased gradually to ∼0.4 g L^-1^ at 1-day SRT (Figure 1). The difference in the VSS between the start-up and SRT-driven phases implies different microbial populations at the two phases.

**FIGURE 2:**
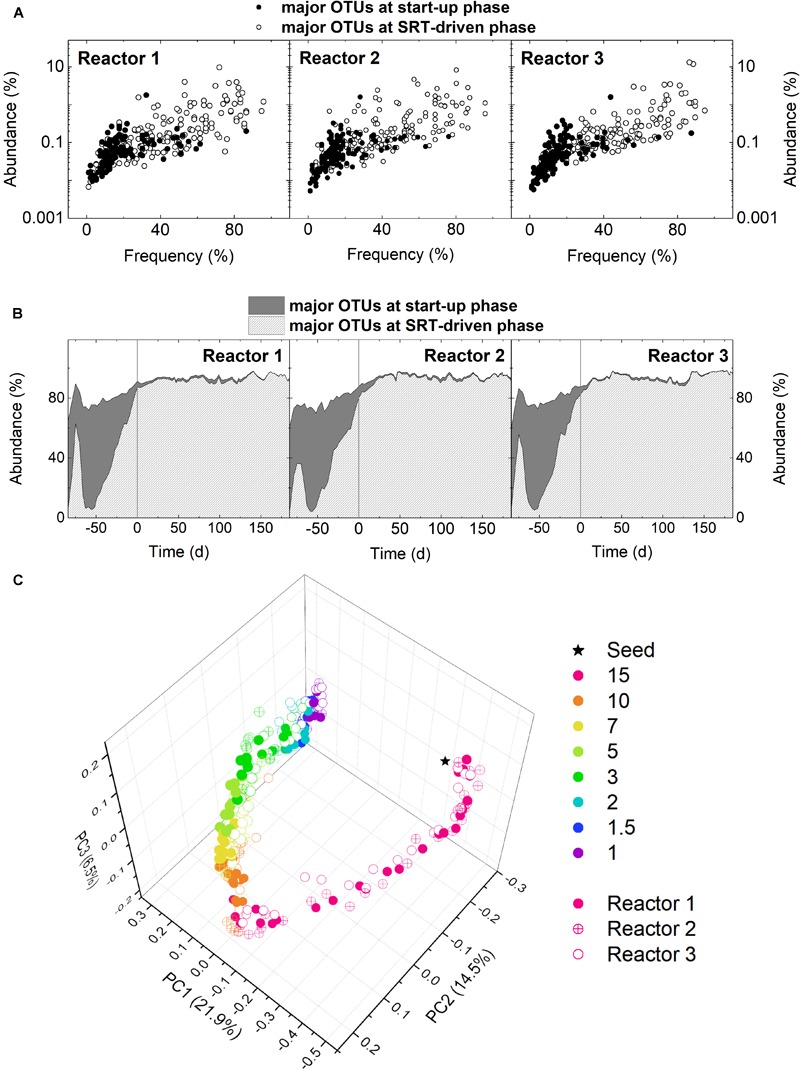
**(A)** Occupancy distribution and **(B)** total abundance of the major OTUs at the start-up and SRT-driven phases in the three reactors. **(C)** PCoA based on unweighted UniFrac.

To detail the change in microbial community structure at the start-up and SRT-driven phases, samples were analyzed using 16S rRNA gene amplicon sequencing. Results of different microbial diversity indices (i.e., Chao1, Shannon, and Simpson) indicated a significant decrease in microbial diversity from the start-up phase to 10-day SRT for all three reactors (Welch’s *t*-test, *p* < 0.05; Supplementary Figure S3). The difference in microbial community structure between the start-up and SRT-driven phases was further demonstrated by examining the frequency and abundance of the major OTUs. An OTU with an abundance ≥0.5% in at least one sample were defined as a major OTU. Based on this criterion, there were 312, 329, and 294 major OTUs identified in Reactors 1, 2, and 3, respectively. More than 40% of the major OTUs were identified only at the start-up phase (Figure 2A). Namely, these OTUs showed an abundance higher than 0.5% at the SRT of the 15 days, but below 0.5% in all samples at the SRTs shorter than 10 days. The total abundance of these OTUs reached the peak of 70% in the middle of the start-up phase, and decreased to <2% at the SRT-driven phase (Figure 2B), suggesting that they were washed out or outcompeted at shorter SRTs. Results of the principle coordinate analysis (PCoA) based on unweighted UniFrac distance also confirmed the changes in microbial community structures at the start-up and SRT-driven phases (Figure 2C). Based on the differences at the two phases, the community assembly mechanisms were discussed separately in the following sections.

### Stochastic Processes Affecting Microbial Succession at the Start-Up Phase

The drastic loss of the major OTUs at the start-up phase (Figure 2B) can be attributed to the prolonged SRT (15 vs. 2.8 days of the WWTP where the seed was collected) and the use of synthetic wastewater (which was prepared with acetate and chloride-containing tap water). This together with the fluctuating VSS (Figure 1) and the clear shift in community structure (Figure 2C) implied primary succession of the microbial communities. To identify the succession steps, hierarchical clustering based on the Kendall’s τ similarity metric was applied (Supplementary Figure S4). The three reactors showed distinct succession steps. Using a cutoff distance of 1.0 in the dendrogram, Reactor 1 exhibited four succession steps (Supplementary Figure S4A). Although Reactor 2 also exhibited four succession steps, the duration of each step was different from that in Reactor 1 (Supplementary Figure S4B). On the other hand, Reactor 3 behaved distinctly from the other two reactors (Supplementary Figure S4C) in the number of succession steps (3 vs. 4). Other cutoffs were also examined, but no consensus on the successional steps could be observed. Given that the reactors were inoculated with identical seed and operated in parallel, the differentiated succession steps indicated a significant role of stochastic processes in the community assembly during primary succession.

### Deterministic Processes Affecting Community Composition at the SRT-Driven Phase

**FIGURE 3:**
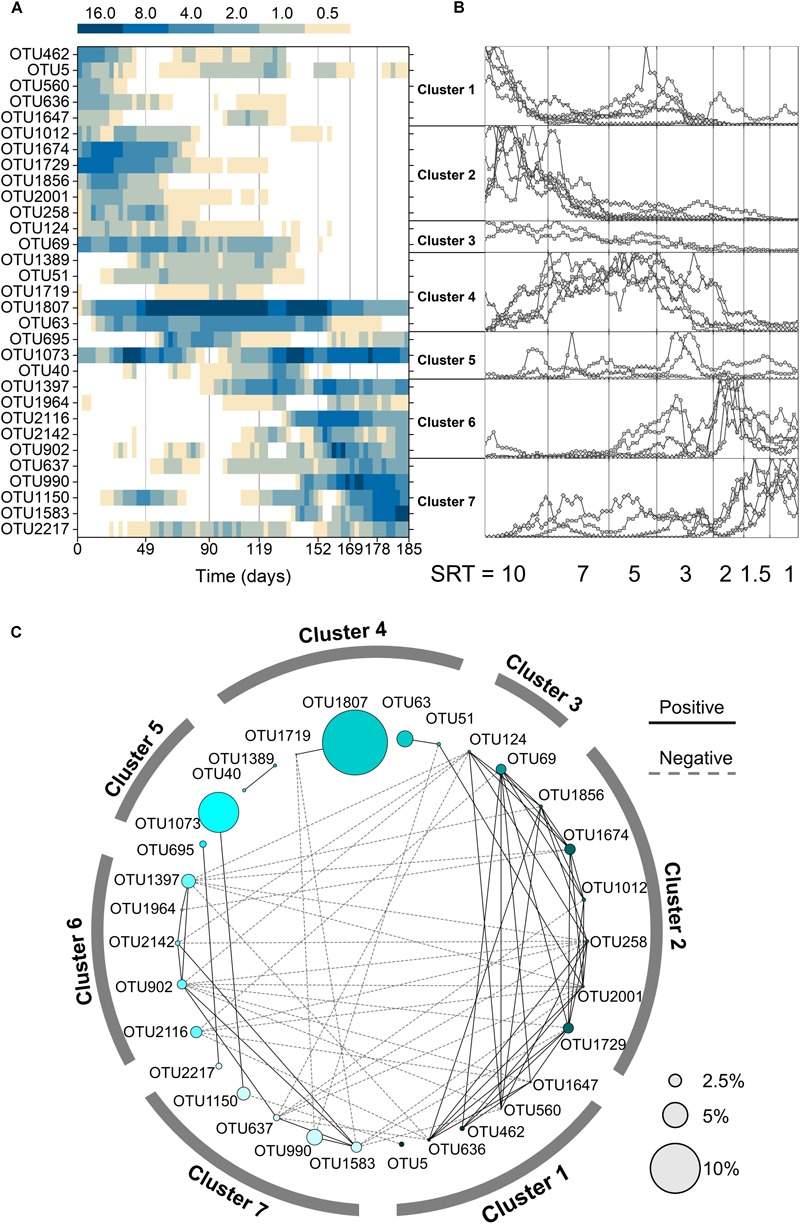
**(A)** Average relative abundance of the 31 shared core OTUs in all three reactors. **(B)** Clustering of the shared core OTUs based on normalization and K-means clustering. The *y*-axis of each cluster represents the ratio normalized to the maximum abundance. **(C)** Network of the shared core OTUs. Node size represents the average relative abundance across all samples and reactors.

To understand the microbial community assembly mechanisms at varied SRTs, core OTUs were selected based on SRT-specific abundance and frequency. At the SRT-driven phase, reactors 1, 2, 3 contained 72, 67, and 56 OTUs as the core populations, respectively, which accounted for over 80% of the total abundance in each reactor. The three reactors shared 31 OTUs (Figure 3A), contributing to ∼65% of the abundance. Among the shared core OTUs, 17 were observed to synchronize in all three reactors: they were pairwise correlated with the Spearman’s rho ranging from 0.43 to 0.85 (*p* < 0.05; Supplementary Table S1). These synchronized core OTUs made up to ∼46% of the total abundance. The sharing and synchrony of the core OTUs in the parallel reactors suggested that the microbial community at the SRT-driven phase was shaped mainly by deterministic processes.

Two deterministic factors, niche differentiation (i.e., SRT) and interspecies interactions (e.g., symbiosis or competition), were both governing the community assembly at the SRT-driven phase. It can be seen from Figure 3B that the occurrence of the 31 core OTUs is strongly associated with SRT and can be divided into three SRT ranges using K-means clustering: long SRTs (10–7 days, cluster 1–3), medium SRTs (5–3 days, cluster 4 and 5), and short SRTs (2–1 day, cluster 6 and 7). Meanwhile, the interspecies interactions of core OTUs were revealed using network analysis (Figure 3C). Positive correlations were observed predominantly within each SRT range, likely because the core population in an SRT range consisted of unique primary and secondary metabolizers that adapted to the specific niche and formed symbiotic relationships. On the other hand, the mutual exclusion occurred mainly between long and short SRT ranges might be because the primary metabolizers in the two SRT ranges competed for acetate.

**FIGURE 4:**
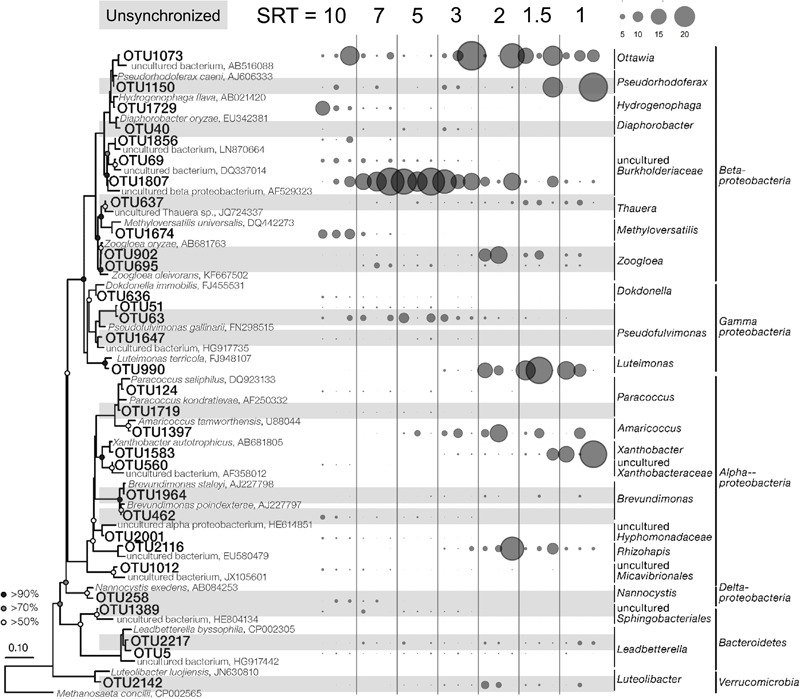
Phylogenetic tree and SRT-specific abundance of the 31 shared core OTUs. The three bubbles under each SRT represent the SRT-specific abundances in Reactor 1, 2, and 3. The shaded OTUs were unsynchronized in the three reactors.

It is reasonable to speculate that the synchronized core OTUs with high abundance and frequent occurrence are primary metabolizers of acetate degradation. For instance, the *Hydrogenophaga*-related OTU 1729 and *Methyloversatilis*-related OTU 1674 were possibly acetate utilizers at long SRTs (Figure 4). OTUs 1807 and 1073 were putative primary metabolizers at medium SRTs (Figure 4), but were not affiliated with known species and their ecological roles warrant further investigation. At short SRTs, OTUs 1397 and 990 were dominant and phylogenetically closed to the genera *Amaricoccus* and *Luteimonas*, respectively (Figure 4). The dominance of these putative primary metabolizers was highly dependent on SRT, possibly due to the differences in growth characteristics such as maximum specific growth rate and substrate affinity. At long SRTs, the VSS was high (Figure 1) and food-to-biomass (F/M) ratio was low, resulting in the selection of K-strategist with low growth rate. As SRT was reduced, VSS decreased and F/M ratio raised, and consequently r-strategists with high growth rate became dominant. The remaining shared core OTUs could be reasoned as secondary metabolizers that degraded intermediates and soluble microbial products generated from the acetate utilizers. As shown in Figure 3C, the positive associations within an SRT range indicate close trophic interactions between the two types of metabolizers.

### Contribution of Stochastic and Deterministic Processes to Community Assembly

The dominance of the shared and synchronized core OTUs in the three reactors is in line with the niche-based theory, and is a clear result of deterministic processes (i.e., changes in SRT and interspecies interactions). However, the microbial community structures among the three reactors differentiated slightly at the SRT-driven phase, indicating the influence of stochastic processes on the microbial assembly. The UPGMA clustering analysis based on unweighted UniFrac distance matrix showed that the sample distance within a given SRT was different among the three reactors (Supplementary Figure S5). Take 10-day SRT as an example, Reactor 1 formed two clusters, whereas Reactor 2 and 3 did not form clear clusters. This difference became more prominent at short SRTs, as supported by the significant differences in α-diversity indices (ANOVA, *p* < 0.05; Supplementary Figure S3) and unweighted UniFrac distance (PERMAVONA, *p* < 0.05) at SRTs shorter than 2 days.

The effects of stochastic processes at the SRT-driven phase could be further reflected by the composition of the core populations. First, within the core OTUs, more than 45% were not shared by all three reactors (41, 36, and 25 OTUs in Reactor 1, 2, and 3, respectively; Supplementary Figure S6), and over 25% were found in only one reactor. These OTUs tended to appear within a narrow range of SRTs and were most likely acting as secondary metabolizers. Second, of the 31 shared OTUs, 14 did not synchronize (Supplementary Table S1). Using OTU 902 at 2-day SRT as an example, the average abundance was 12.5 and 17.0% in Reactor 1 and 2, respectively, but only 0.5% in Reactor 3 (Figure 4). The unshared and unsynchronized OTUs together contributed to 28–42% of the total abundance in the three reactors. Overall, the results suggest that stochastic processes play an important role in the microbial assembly at the SRT-driven phase.

**FIGURE 5:**
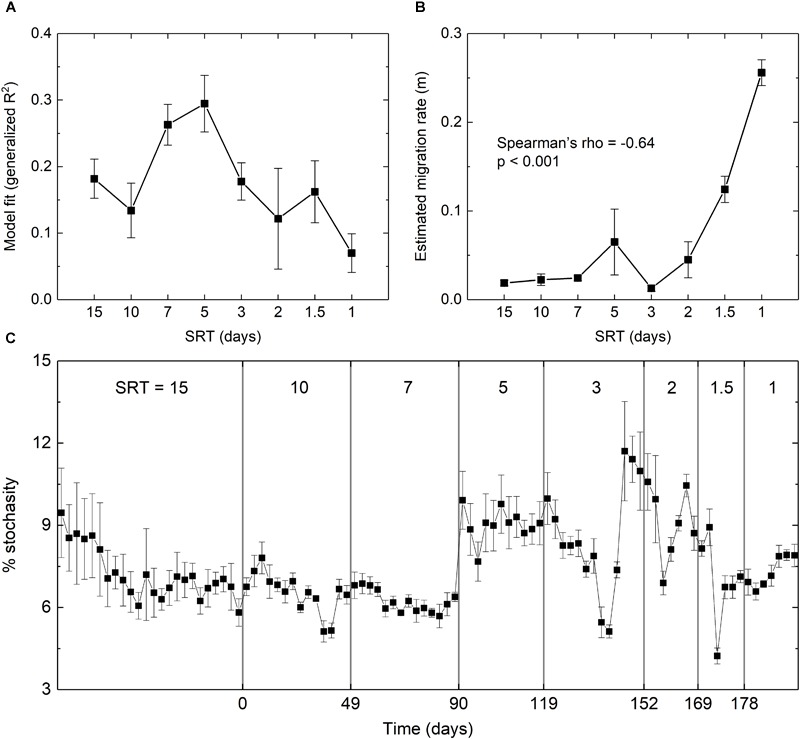
**(A)** Goodness-of-fit and **(B)** estimated migration rate of the fit using Sloan’s neutral model. **(C)** Percentage of stochasticity calculated using the null model. Error bars were calculated based on the simulation results of the three reactors.

The extent to which stochastics drives microbial assembly at the start-up and SRT-driven phases was estimated using Sloan’s Neutral Community Model. As shown in Figure 5A, the fit of the model was significantly improved from *R*^2^ = 0.18 at 15-day SRT to *R*^2^ = 0.29 at 5-day SRT, but gradually dropped to *R*^2^ = 0.07 at 1-day SRT. The estimated migration rate (*m*) represents the probability that a random loss of an OTU at a given SRT in a reactor is replaced by dispersal from the meta-community across all SRTs. It was negatively correlated with the SRT (Spearman’s rho = -0.64, *p* < 0.001, Figure 5B) and reached the peak of 0.26 at the shortest SRT, indicating that stochastic processes such as drift and dispersal were increasingly important as the selection pressure imposed by SRT became stronger.

The null model analysis further confirmed the intensified stochasticity at shorter SRTs (Figure 5C). During the star-up phase, the relative importance of stochasticity to community assembly decreased from 9 to 6%, suggesting an increased contribution of deterministic processes. During the SRT-driven phase, stochasticity remained relatively low at the SRTs of 10 and 7 days, and increased at the SRTs of 5 days or shorter. The percentage of stochasticity at SRT ≤ 2 days was significantly higher than that at SRT ≥ 7 days (Welch’s *t*-test, *p* < 0.05). The PERMDISP results showed that the actually community and the null assemblage were significantly different across all SRTs for the triplicate reactors (*p* < 0.001), indicating that the community dynamics was mainly governed by deterministic processes. This was expected due to the strong selection pressure imposed by SRT.

## Discussion

Various microbial processes have been developed in engineering systems to mimic the important microbial functions (e.g., nutrient removal, biogas production, etc.) occurring in nature to treat domestic and industrial waste streams (Grady, Daigger et al., 2011). To establish the desired microbial communities, an acclimation or start-up phase is required prior to optimizing operation conditions. During this procedure, stochastic and deterministic processes are identified as two major mechanisms shaping community structure (Ofiţeru et al., 2010), but it remains unclear to what extent stochastic and deterministic processes interact with each other and contribute to the selection. The present study addressed this question by characterizing the microbial communities in three lab-scale activated sludge reactors under a strong deterministic factor (i.e., SRT).

In this study, the three reactors were fed with acetate as a readily degradable substrate. At the start-up phase under a long SRT of 15 days, microbial succession was observed (Supplementary Figure S4). Many studies have investigated the microbial community succession in different ecosystems and reported that the succession is driven predominantly by deterministic factors (Koenig et al., 2011; Podell et al., 2013; Datta et al., 2016; Lu et al., 2016). However, the operation conditions of the activated sludge reactors used here were less deterministic than in previously reported, and thus stochastic processes were likely playing an important role in structuring the community assembly. This can be explained by a conceptual model (Dini-Andreote et al., 2015) that the microbial succession was initially governed by stochastic processes, and as the microbes altered their environment, the community was progressively shaped by interspecies interactions. The impacts of stochastic processes were demonstrated by the differentiated successional steps observed among the three reactors (Supplementary Figure S4). The shift from stochastic to deterministic processes was also confirmed by the decreasing contribution of stochasticity in the null model analysis (Figure 5C). Collectively, the community assembly during the start-up phase was affected both by stochastic and deterministic processes.

At the SRT-driven phase, the community assembly was governed mainly by deterministic factors such as niche differentiation (i.e., varied SRT) and interspecies interactions (i.e., competition for acetate and cross-feeding between the primary and secondary metabolizers). As the SRTs were sequentially reduced, a decrease in VSS (Figure 1) and a clear shift in community structure (Figure 3) were observed, leading to an increased F/M ratio. The results indicated the selection of slow-growing K-strategists at low F/M ratio and fast-growing r-strategists at high F/M ratio (Andrews and Harris, 1986). It is reasonable to assume that the dominant K- and r-strategists at the respective SRT ranges are responsible for acetate utilization. These two types of primary metabolizers had different growth kinetics in acetate metabolisms that allowed them to dominate at different SRTs. For example, OTU 1807, which could be considered as a K-strategists, was abundant at medium SRT range (Figure 3). Based on the MiDAS database (Mcilroy et al., 2017), this OTU is found to be phylogenetically close to the genus *Trichococcus*, an aerobic heterotrophic generalist that have been widely found in activated sludge communities (Vandewalle et al., 2012; Saunders et al., 2016). On the other hand, OTU 1397 as a r-strategist was abundant at short SRTs. It was phylogenetically related to *Amaricoccus* spp., which had also been observed in WWTPs around the world (Maszenan et al., 1997).

Findings of this study including the diversity indices, the composition of the core populations, and the modeling results all suggested that stochastic processes could exert significant effects on the community assembly at the SRT-driven phase. These supported our hypothesis that stochastic processes could be intensified by deterministic processes and the two processes could drive community assembly in an interactive way. A conceptual model predicts that when a deterministic disturbance occurs, a community will return to a pristine condition where all members having equal chance to grow, and consequently stochastic processes become the main driving force of the community assembly (Dini-Andreote et al., 2015). In the present study, the interactions between stochastic and deterministic processes under shorter SRT could be explained by increased frequency of migration (i.e., higher volume of waste sludge discarded from the reactors), as evidenced by the higher estimated migration rate at shorter SRTs (Figure 5B). Such an interactive influence is proposed for the first time in engineered biological systems, and remains to be investigated in large-scale bioreactors.

Although it has been widely accepted that stochastic and deterministic processes occur simultaneously during microbial community formation, disentangling their relative influence is still a compelling challenge facing microbial ecologists. By far, three major approaches have been developed to quantify the importance of ecological stochasticity: multivariate analysis, neutral-theory-based process models, and null modeling analysis (Zhou and Ning, 2017). Variation partitioning analysis as one type of multivariate analysis has been applied to explain the different communities formed in parallel microbial electrolysis cells operated under identical conditions (Zhou et al., 2013). In contrast to microbial electrolysis cells, the activated sludge reactors used here lack deterministic variables such as pH, gas composition and electricity generation, and thus a multivariate analysis may lead to biased results. Instead, we used neutral-theory-based modeling and null modeling to understand the relative influence (Zhou et al., 2014; Burns et al., 2015). The results not only quantified the contribution of stochastic and deterministic processes to community establishment, but also confirmed the interactions between the two processes (Figure 5), presenting a powerful tool to understand microbial community assembly mechanisms.

It should be noted that acetate was used as the main carbon source in this study, and thus the microbial communities were shaped toward acetate metabolism. In contrast, real wastewater contains a variety of substrates, and the communities in full-scale WWTPs are composed of not only generalists (e.g., *Trichococcus*-related OTU 1807), but also diverse specialist guilds, including nitrifier, phosphate accumulating organisms, hydrolyzers, bulking and foaming bacteria, etc. It has been proposed that generalists are more likely to be assembled randomly, whereas specialists are selected mainly by deterministic factors (Liao et al., 2016). The deterministic selection of specialists has been demonstrated in an activated sludge community, where the metabolic potential of micropollutant removal is impaired with decreased SRT (Vuono et al., 2016a,b). These observations suggest that, in order to better understand the assembly mechanisms in complicated communities, the interactive effects of stochastic and deterministic processes on different functional guilds should be quantified separately.

Microbial immigration from sewers to WWTPs is another critical process to be considered when studying community assembly (Frigon and Wells, 2019). Immigrating microbes may replace indigenous microbes in the receiving community, especially after disturbance, thereby promoting random community assembly. For example, the percentage of OTUs shared by activated sludge and influent was found to be less than 10% under low selection pressure (SRT = 10.5 days) (Lee et al., 2015), but was as high as 62% under high selection pressure (SRT = 3 days) (Callahan et al., 2016). The results can be explained using the abovementioned conceptual model (Dini-Andreote et al., 2015), which describes that disturbance indiscriminately eliminates resident populations in a community and creates niche space for immigrants to colonize. The implication that stochastic processes can be further intensified in the presence of both strong deterministic factors and constant microbial immigration is of fundamental and practical significance to understand the interplay between these mechanisms and warrant further study.

## Data Availability

The datasets generated for this study can be accessed from the NCBI GenBank, PRJNA478774.

## Author Contributions

All authors conceived and designed the study, revised the manuscript, and read and approved the submitted version of the manuscript. JL and RM conducted the experiments. HY performed the data analysis and wrote the first draft of the manuscript.

## Conflict of Interest Statement

The authors declare that the research was conducted in the absence of any commercial or financial relationships that could be construed as a potential conflict of interest.
