# Supplementary material for: Nexus of Stochastic and Deterministic Processes on Microbial Community Assembly in Biological Systems
Source: Front Microbiol. 2019 Jul 4;10:1536. doi: 10.3389/fmicb.2019.01536 (PMC6621641; doi:10.3389/fmicb.2019.01536)
Supplement: Supplementary file 1 [file Data_Sheet_1.docx]

Supplementary Material

R script for Neutral model analysis

**## NEUTRAL MODEL, adapted from Burns et al. ISME J. 2015**

# The following script is for MLE fitting in one reactor. Fitting was performed for each reactor.

# Dependence

library(minpack.lm)

library(Hmisc)

library(stats4)

# Import following parameters

# pool: A community table for all SRTs with samples as rows and taxa as columns. All samples must be rarefied to the same depth.

# spp: A community table for one specific SRT with samples as rows and taxa as columns.

# Calculate the number of individuals per community

N <- mean(apply(pool, 1, sum))

# Calculate the average relative abundance of each taxon across communities

p.m <- apply(pool, 2, mean)

p.m <- p.m[p.m != 0]

p <- p.m/N

# Calculate the occurrence frequency of each taxon at the specific SRT

spp.bi <- 1*(spp>0)

freq <- apply(spp.bi, 2, mean)

freq <- freq[freq != 0]

# Combine

C <- merge(p, freq, by=0)

C <- C[order(C[,2]),]

C <- as.data.frame(C)

C.0 <- C[!(apply(C, 1, function(y) any(y == 0))),] # Removes rows with any zero (absent in either source pool or local communities)

p <- C.0[,2]

freq <- C.0[,3]

names(p) <- C.0[,1]

names(freq) <- C.0[,1]

# Calculate the limit of detection

d = 1/N

# Fit model parameter m (or Nm) using Non-linear least squares (NLS)

m.fit <- nlsLM(freq ~ pbeta(d, N*m*p, N*m*(1-p), lower.tail=FALSE), start=list(m=0.1))

m.ci <- confint(m.fit, 'm', level=0.95)

m[n,i] <- coef(m.fit)

# Calculate goodness-of-fit (R-squared and Root Mean Squared Error)

freq.pred <- pbeta(d, N*coef(m.fit)*p, N*coef(m.fit)*(1-p), lower.tail=FALSE)

Rsqr[n,i] <- 1 - (sum((freq - freq.pred)^2))/(sum((freq - mean(freq))^2))

RMSE <- sqrt(sum((freq-freq.pred)^2)/(length(freq)-1))

pred.ci <- binconf(freq.pred*nrow(spp), nrow(spp), alpha=0.05, method="wilson", return.df=TRUE)

**## NEUTRAL MODEL, Zhou et al. PNAS 2013**

# Dependence

library(dplyr)

library(dendextend)

library(vegan)

# Import the OTU tables of the three reactors (otu1, otu2, otu3), row is sample, column is taxon

# Calculate the richness of each sample

rich1 = apply(1*(otu1>0), 1, sum)

rich2 = apply(1*(otu2>0), 1, sum)

rich3 = apply(1*(otu3>0), 1, sum)

# Calculate the expected and actual Jaccard's between Reactor 1&2. The process is repeated for Reactor 2&3 and Reactor 3&1.

J.exp <- vector()

J.obs <- vector()

null <- data.frame()

expect <- data.frame()

for(n in 1:96) # 96 samples

{

sample1 <- otu1[n,] # assign sample to be tested

sample2 <- otu2[n,]

J <- vector()

for(m in 1:999) # repeat random drawing

{

p1 <- sample1[1,sample(ncol(sample1), rich1[n], replace=FALSE)] # subsampling of Reactor 1 based on the richness of sample n

p2 <- sample2[1,sample(ncol(sample2), rich2[n], replace=FALSE)]

C <- bind_rows(p1, p2) # bind two subsamples

C[is.na(C)] <- 0

J[m] <- jaccard(C[1,], C[2,]) # expected Jaccard's similarity

null <- bind_rows(null, p1) # collect the subsample of Reactor 1 at each time point

}

J.exp[n] <- mean(J) # mean expected Jaccard's similarity

J.obs[n] <- jaccard(sample1, sample2) # actuall Jaccard's similarity

null[is.na(null)] <- 0

expect <- bind_rows(expect, round(apply(null,2,mean),0)) # construct the null community of reactor 1

}

# Calculate %stochasity between Reactor 1&2

SS = 1 - (J.obs - J.exp)/J.obs

# PERMDISP between the actual and null community of Reactor 1

all <- bind_rows(otu1, expect)

dis <- vegdist(all, method = "jaccard", na.rm = T)

groups <- factor(c(rep(1,nrow(all)/2), rep(2,nrow(all)/2)), labels = c("actual", "null"))

mod <- betadisper(dis, groups)

permutest(mod, pairwise = TRUE)

**Supplementary Table 1.** Pairwise Spearman correlation of the 31 shared core OTUs in the triplicate reactors. Unsynchronized OTUs with Spearman’s rho <0 and p-value >0.05 are highlighted.

|  | Reactor 1 & 2 | | Reactor 2 & 3 | | Reactor 3 & 1 | |
| --- | --- | --- | --- | --- | --- | --- |
|  | rho | p | rho | p | rho | p |
| ***Synchronized*** |  |  |  |  |  |  |
| OTU124 | 0.625 | 0 | 0.293 | 0.015 | 0.391 | 0.001 |
| OTU51 | 0.486 | 0 | 0.655 | 0 | 0.406 | 0.003 |
| OTU5 | 0.258 | 0.032 | 0.599 | 0 | 0.443 | 0 |
| OTU1012 | 0.632 | 0 | 0.463 | 0 | 0.466 | 0 |
| OTU1073 | 0.646 | 0 | 0.669 | 0 | 0.509 | 0 |
| OTU1807 | 0.622 | 0 | 0.746 | 0 | 0.541 | 0 |
| OTU636 | 0.777 | 0 | 0.705 | 0 | 0.652 | 0 |
| OTU990 | 0.935 | 0 | 0.786 | 0 | 0.737 | 0 |
| OTU1583 | 0.643 | 0 | 0.578 | 0.001 | 0.739 | 0 |
| OTU1397 | 0.732 | 0 | 0.355 | 0.015 | 0.757 | 0 |
| OTU2116 | 0.832 | 0 | 0.895 | 0 | 0.783 | 0 |
| OTU69 | 0.649 | 0 | 0.435 | 0 | 0.803 | 0 |
| OTU560 | 0.656 | 0 | 0.694 | 0 | 0.815 | 0 |
| OTU1674 | 0.737 | 0 | 0.850 | 0 | 0.840 | 0 |
| OTU1856 | 0.845 | 0 | 0.731 | 0 | 0.843 | 0 |
| OTU2001 | 0.760 | 0 | 0.644 | 0 | 0.889 | 0 |
| OTU1729 | 0.864 | 0 | 0.801 | 0 | 0.898 | 0 |
|  |  |  |  |  |  |  |
| ***Unsynchronized*** |  |  |  |  |  |  |
| OTU1389 | -0.217 | 0.249 | 0.606 | 0.001 | 0.048 | 0.765 |
| OTU902 | 0.679 | 0 | 0.114 | 0.476 | 0.08 | 0.602 |
| OTU695 | 0.529 | 0 | 0.106 | 0.505 | 0.087 | 0.583 |
| OTU637 | 0.491 | 0.003 | 0.381 | 0.001 | 0.108 | 0.536 |
| OTU2142 | 0.678 | 0 | -0.325 | 0.005 | 0.167 | 0.166 |
| OTU1964 | 0.320 | 0.009 | 0.043 | 0.761 | 0.236 | 0.1 |
| OTU1150 | 0.520 | 0 | -0.15 | 0.305 | 0.227 | 0.075 |
| OTU1647 | 0.094 | 0.511 | 0.191 | 0.158 | -0.324 | 0.022 |
| OTU2217 | 0.154 | 0.209 | 0.044 | 0.732 | 0.325 | 0.009 |
| OTU258 | -0.264 | 0.235 | -0.663 | 0.003 | -0.652 | 0.001 |
| OTU40 | 0.748 | 0 | 0.405 | 0.120 | 0.713 | 0.001 |
| OTU462 | 0.449 | 0.001 | 0.174 | 0.269 | 0.606 | 0 |
| OTU63 | 0.693 | 0 | 0.059 | 0.625 | 0.673 | 0 |
| OTU1719 | 0.513 | 0.002 | -0.174 | 0.278 | 0.736 | 0 |


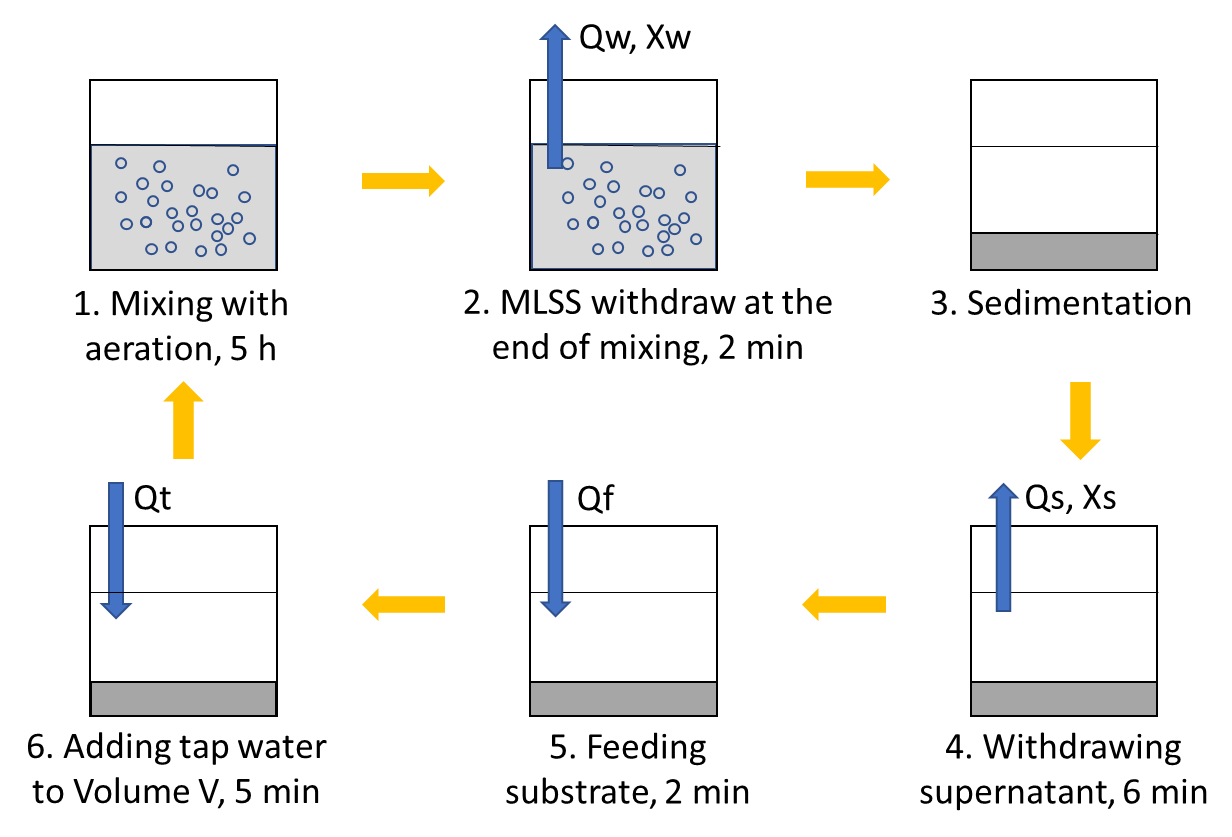


**Supplementary Figure S1.** Schematics of experimental setup.


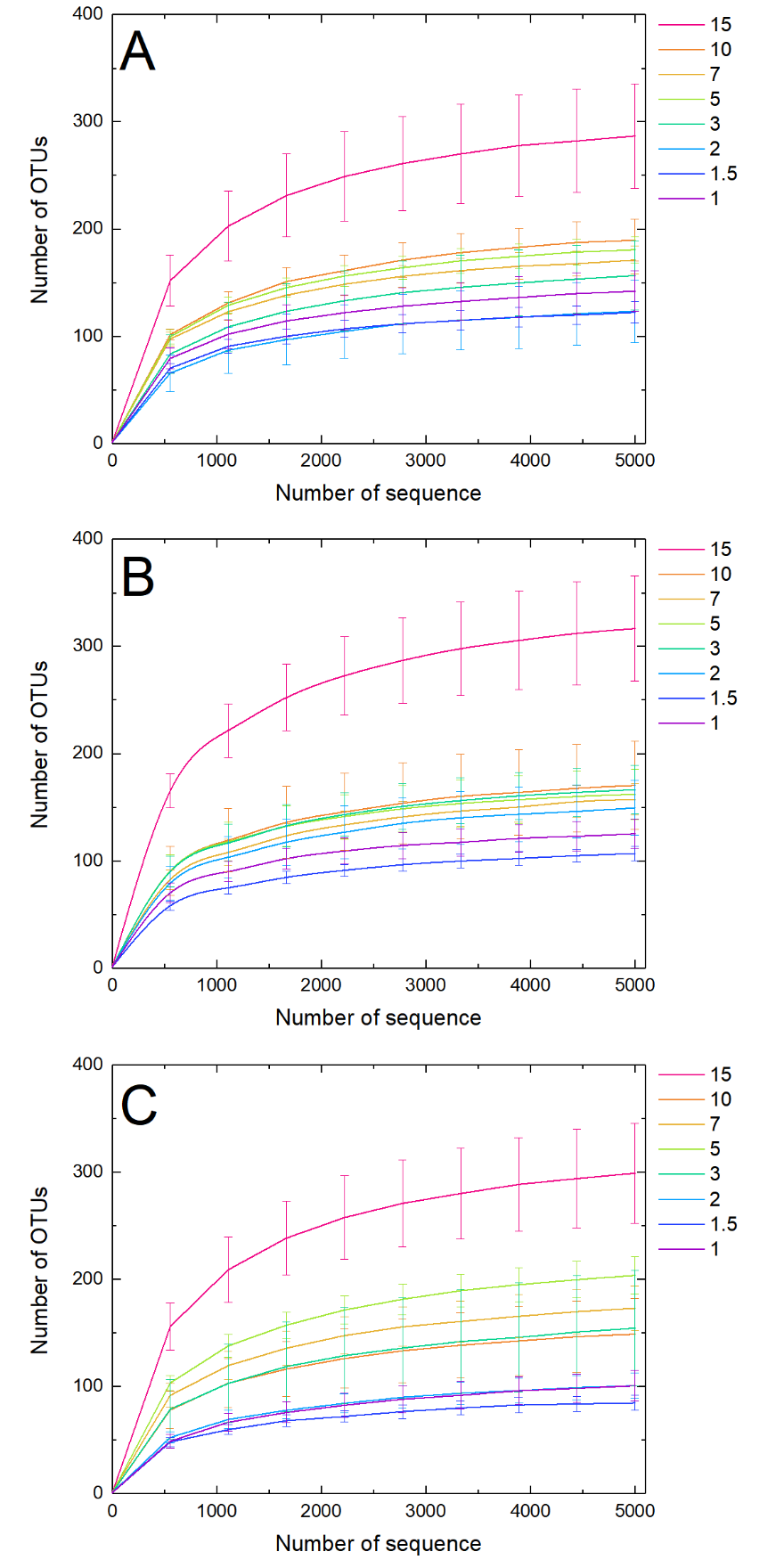


**Supplementary Figure S2.** Rarefaction curves of the communities at different SRTs in (A) Reactors 1, (B) Reactors 2 and (C) Reactors 3.


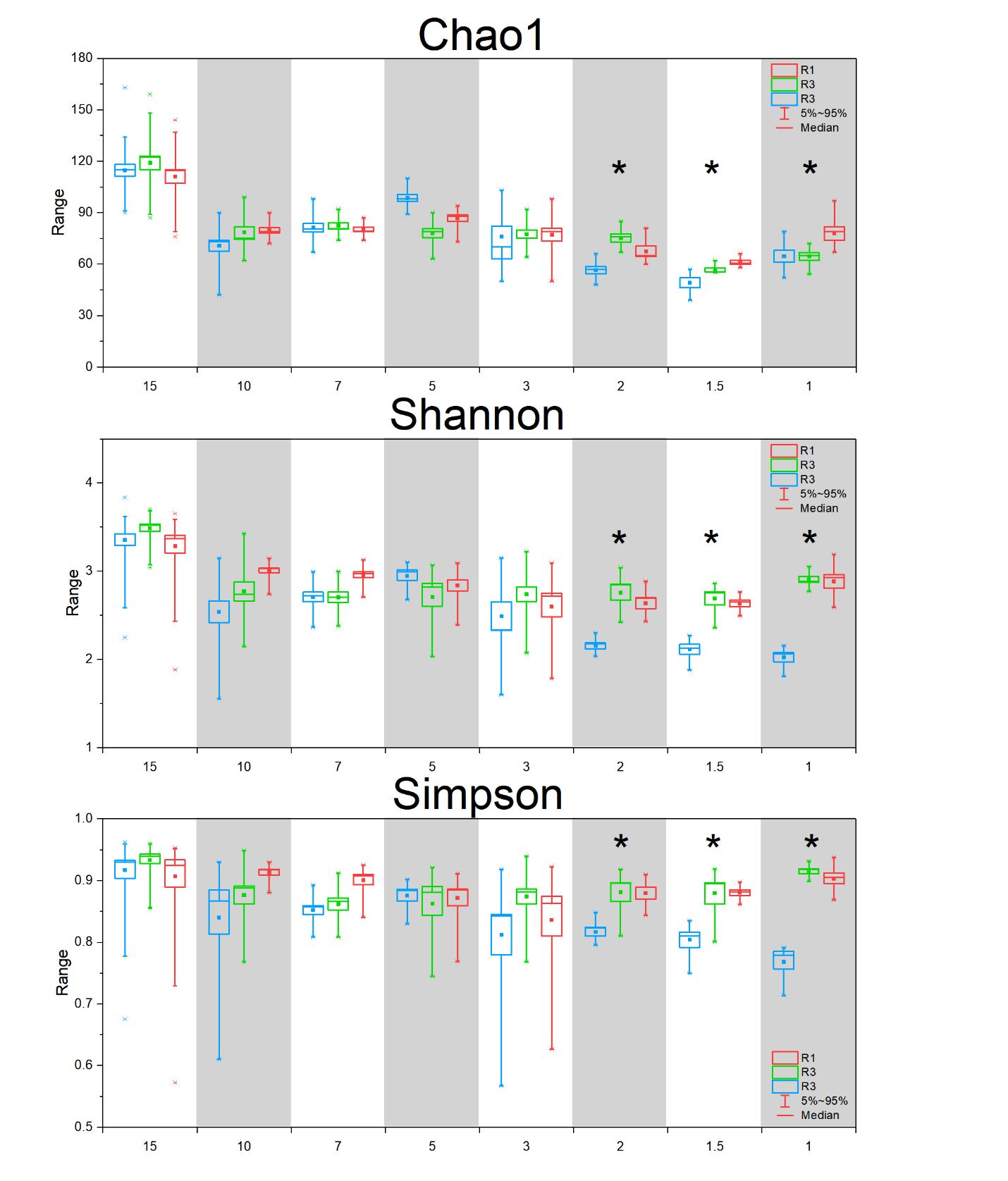


**Supplementary Figure S3.** Alpha-diversity indices of the communities at different SRTs in the three reactors. Asterisk indicates significant difference among the reactors (ANOVA, p < 0.05).


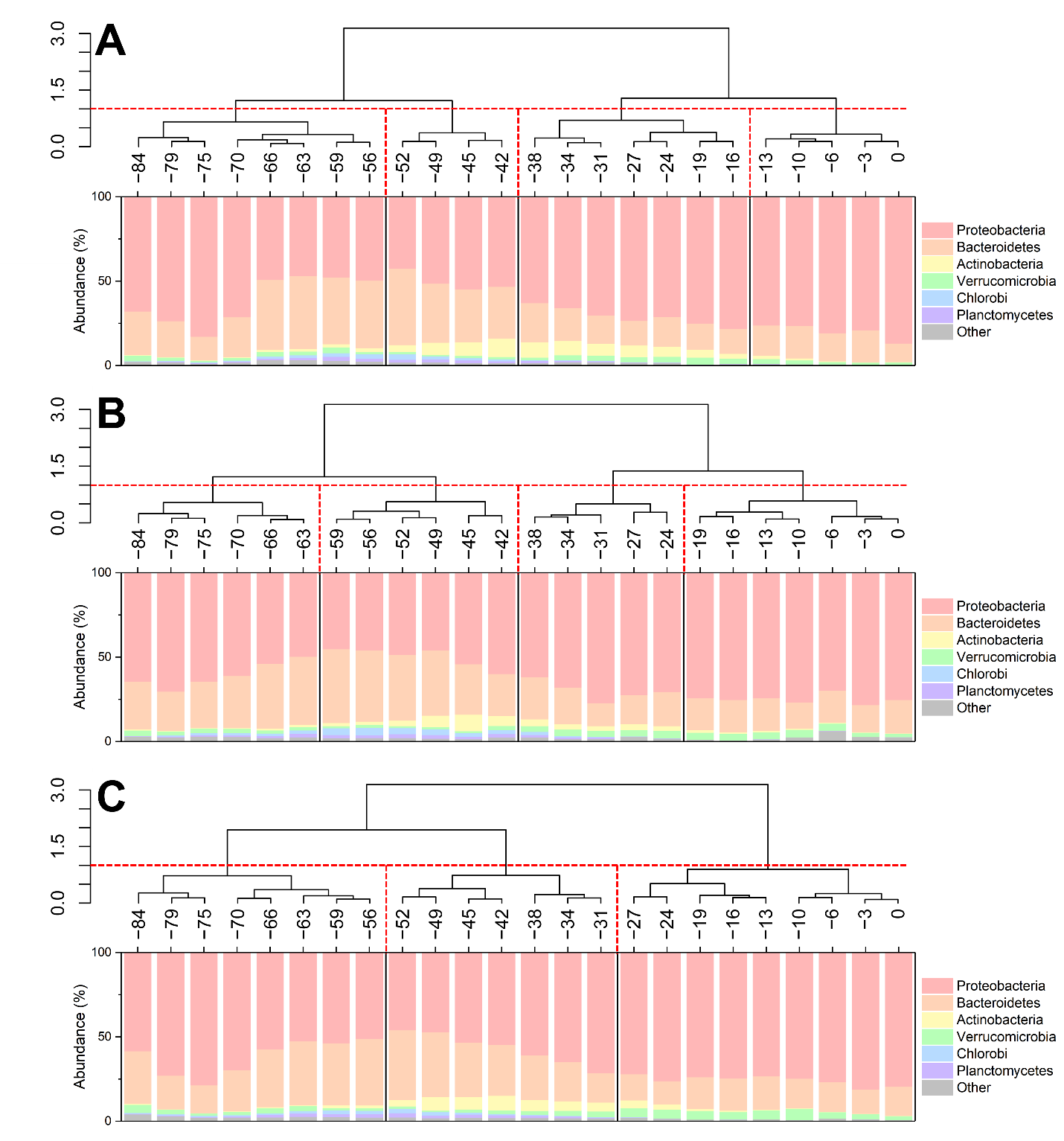


**Supplementary Figure S4.** Hierarchical clustering (based on the Kendall’s τ similarity metric of the major OTUs at 15-d SRT) and the relative abundance of the dominant phyla at the start-up phase in (A) Reactors 1, (B) Reactors 2 and (C) Reactors 3.


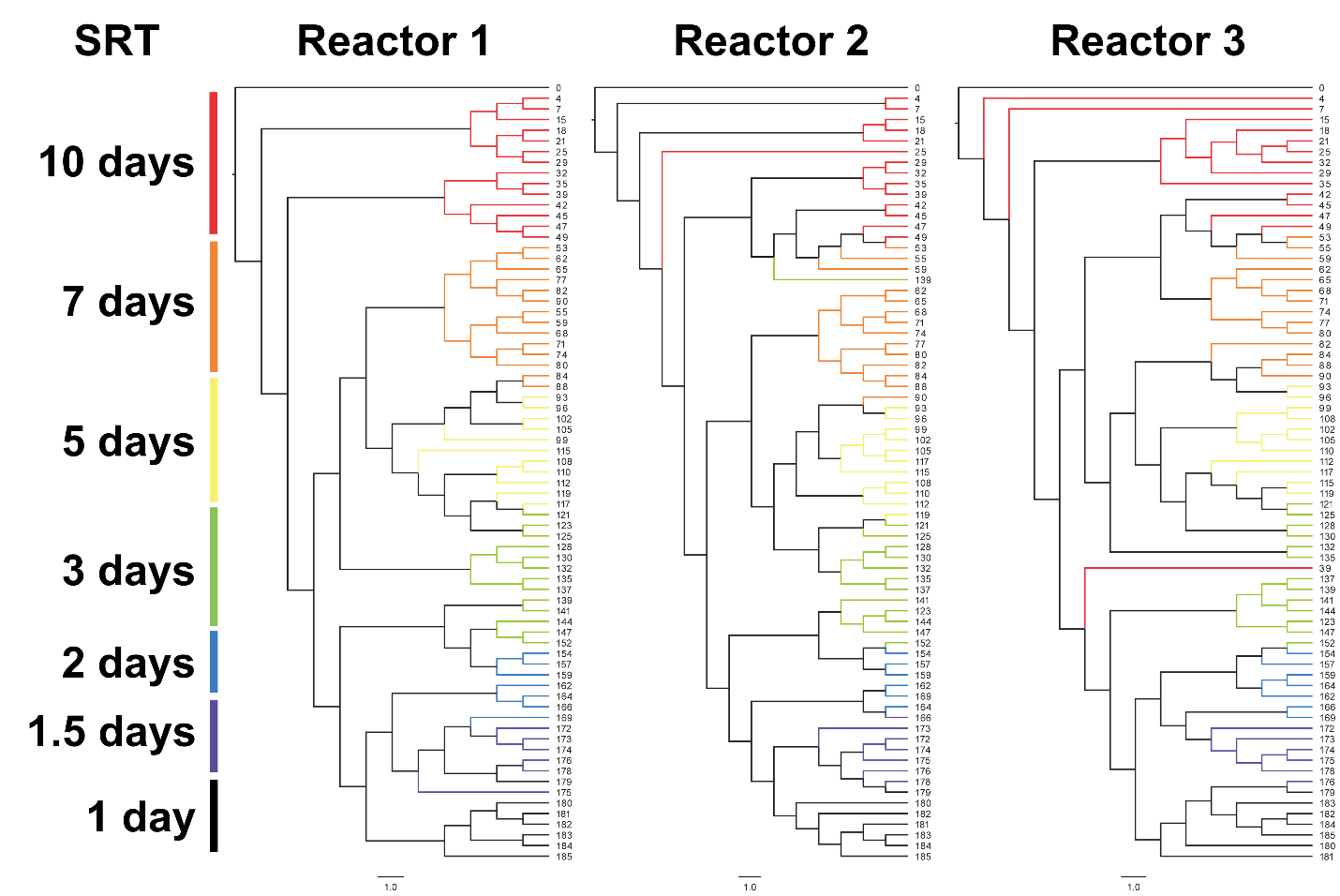


**Supplementary Figure S5.** UPGMA dendrogram built based on unweighted UniFrac distance matrix and the sample on day 0 as the root.


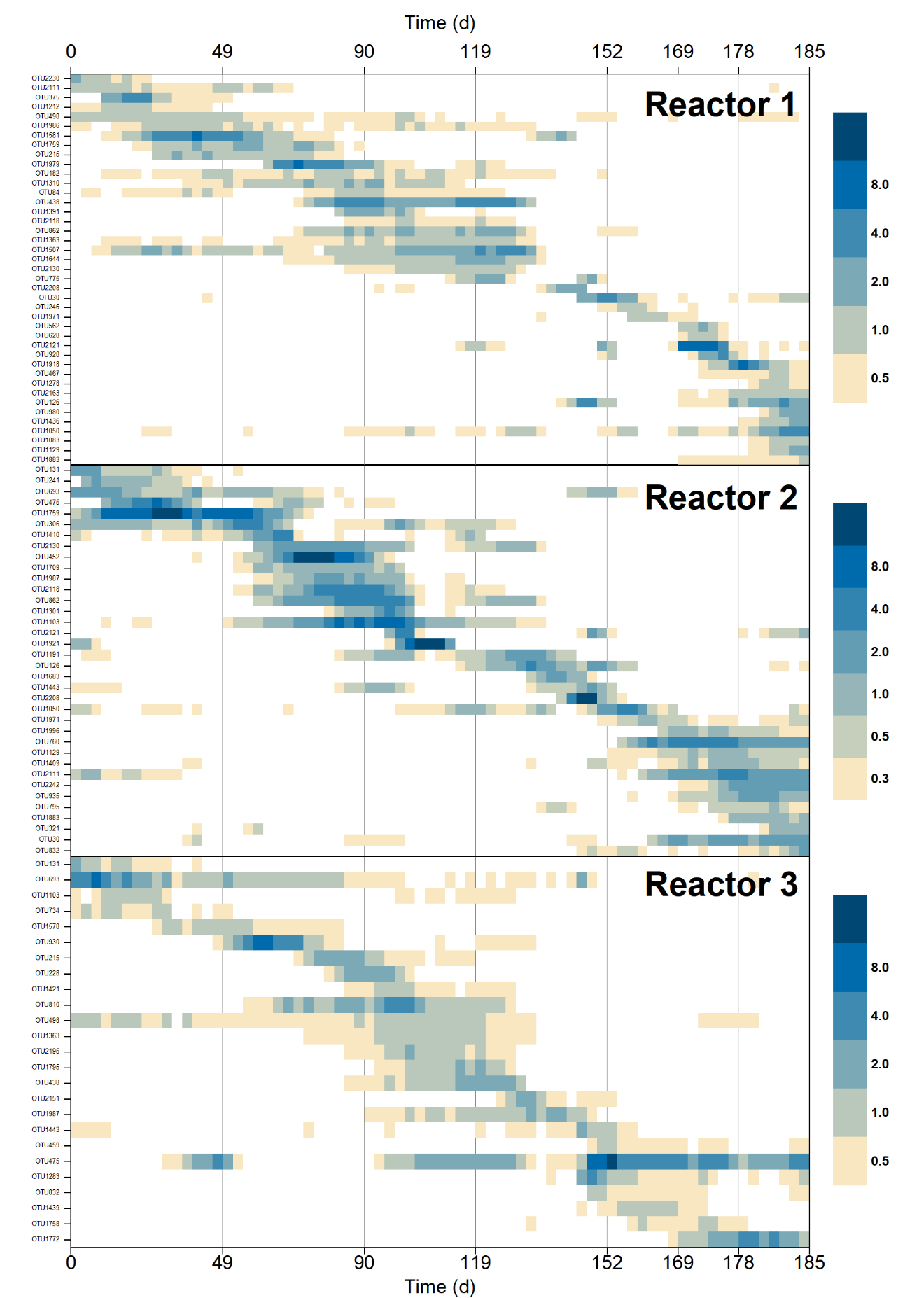


**Supplementary Figure S6.** Relative abundance of the unshared core OTUs in the three reactors.
